# Feasibility of Audio-Recording Consultations with Pregnant Australian Indigenous Women to Assess Use of Smoking Cessation Behaviour Change Techniques

**DOI:** 10.1155/2021/6668748

**Published:** 2021-01-13

**Authors:** Yael Bar-Zeev, Eliza Skelton, Michelle Bovill, Maree Gruppetta, Billie Bonevski, Gillian S. Gould

**Affiliations:** ^1^School of Medicine and Public Health, The University of Newcastle, Callaghan, New South Wales, Australia; ^2^Braun School of Public Health and Community Medicine, The Hebrew University of Jerusalem-Hadassah Medical Organization, Israel

## Abstract

**Introduction:**

Behavioural counselling is an effective method to improve smoking cessation during pregnancy. Audio recordings of consultations have been used previously to assess fidelity in specialized smoking cessation services, but not in primary care.

**Aims:**

The study is aimed at assessing the feasibility of audio-recording smoking cessation counselling as part of an intervention in primary care settings and exploring the number and type of behaviour change techniques (BCTs) delivered.

**Methods:**

This study was a nested feasibility study within a larger trial. Health providers (HPs) and pregnant women were asked to agree or decline audio recording their smoking-related consultations. Data collected included percentage providing consent, number of recordings performed, HP type, and date (pre/post intervention). Interviews were conducted to assess the trial procedures' acceptability.

**Results:**

Two services provided seven recordings, all pre-intervention. Of the 22 recruited women, 14 consented to being audio recorded (64%) and five provided recordings; of the 23 recruited HPs, 16 agreed (69%), and two provided recordings. Qualitative data suggest that HPs found audio recording difficult to remember. HPs spent on average two minutes discussing smoking and used few BCTs.

**Conclusions:**

Audio recordings of smoking-related counselling were not feasible as planned. Future research will need to explore acceptable methods to assess BCT use in primary care.

## 1. Introduction

Tobacco smoking during pregnancy remains a global public health issue, specifically among disadvantaged populations such as minority groups, those with low socioeconomic status, and Indigenous populations [1]. Smoking rates among Australian Aboriginal and Torres Strait Islander pregnant women significantly declined in recent years, but are still threefold higher than the general population (42% versus 11% in 2016) [2].

Behavioural counselling is an effective method to improve smoking cessation rates during pregnancy [3]. A meta-analysis of 30 studies found that counselling increased smoking cessation rates by 44% (RR 1.44, 95% CI 1.19 to 1.73) [3]. Clinical guidelines recommend primary care health providers (HPs) provide brief behavioural counselling [4, 5]. Guidelines sometimes use a broad term such as “assist with counselling” without further details. Most smoking cessation interventions do not provide a full description of what was included as part of counselling.

Recent novel research has tried to articulate the “active ingredients” of behavioural counselling, defined as behaviour change techniques (BCTs). A BCT is defined as an observable and replicable component designed to change behaviour [6], for example, “instruction on how to perform a behaviour.” A BCT taxonomy has been developed and validated, which includes several categories of BCTs such as those that pertain to goals and planning, feedback and monitoring, social support, repetition and substitution, and regulation and more [6].

A specific taxonomy for smoking cessation has also been developed which includes forty-three possible BCTs [7]; several of them have been suggested as “evidence based” (defined as included in at least two interventions found to be effective in randomized controlled studies and/or associated with biochemically validated abstinence) [8, 9]. A few studies focused specifically on BCTs for smoking cessation in pregnancy, identifying 23 possibly effective BCTs [10, 11]. It is not yet clear which BCT combinations are effective in what context, setting, and subpopulations.

Understanding which BCTs are actually used during smoking cessation consultations can help guide future interventions. Research on smoking cessation behavioural interventions has focused on coding BCTs based on their description in published manuscripts or intervention manuals [10, 11]. A major limitation is fidelity, as it is not always clear whether BCTs were performed as instructed, impacting also the ability to assess the mechanism through which an intervention is effective or not [10, 11]. Identifying actual BCT use can provide objective data for this purpose. Few studies have recorded consultations to analyse which BCTs were employed; those that did were in specialized smoking cessation services [8, 12]. Analysis of recorded consultations has not yet occurred in primary care settings, nor been used to identify effective BCTs for smoking cessation among Indigenous people, including pregnant women.

The aim of this study was to assess the feasibility of audio recording HP-pregnant women's smoking cessation behavioural counselling as part of an intervention in a primary care setting. A secondary aim was to conduct a preliminary exploration of the number and type of BCTs delivered as part of smoking cessation support to pregnant Aboriginal women.

## 2. Materials and Methods

### 2.1. Design

The study was a nested feasibility study within a larger intervention trial—the Indigenous Counselling and Nicotine (ICAN) Quit in Pregnancy [13–15]. The ICAN Quit in Pregnancy trial is aimed at testing the feasibility of an intervention to improve HP smoking cessation care with pregnant Aboriginal and Torres Strait Islander women who smoke in partnership with Aboriginal Medical Services [13–15]. The intervention included an educational resource package, webinar training, and free nicotine replacement therapy. Both the training and the educational resource package put an emphasis on specific BCTs which could support pregnant women to quit smoking, such as goal setting, problem solving, and boosting self-efficacy [13]. As part of the ICAN Quit in Pregnancy trial, both HPs and pregnant women were recruited and followed (November 2016 to September 2017). HPs were asked to complete a pre- and post-intervention survey. Pregnant women were asked to complete surveys and undergo breath carbon monoxide measurements (for smoking status biochemical validation) at baseline and 4-week and 12-week follow-up visits. In each service, one health professional (who also saw pregnant women as part of their routine role) was nominated by the service as a research facilitator (and received additional training regarding research procedures, on top of the smoking cessation support training provided to all HPs). The research facilitator coordinated all of the ICAN Quit in Pregnancy trial components within the service, including recruiting patients, conducting the surveys and evaluations, and collecting the feasibility data. A full description of the ICAN Quit in Pregnancy trial methodology and results has been previously published, including adherence to the CONSORT guidelines [13–15].

### 2.2. Aboriginal Advisory Panel

The ICAN Quit in Pregnancy trial was developed collaboratively with two Aboriginal Medical Services [16]. A Stakeholder and Consumer Aboriginal Advisory Panel guided the development and implementation of this study to ensure Aboriginal community ownership and cultural sensitivity. At least one member from each Aboriginal Medical Service was invited to participate on this panel [16]. Full details can be found elsewhere [13, 14]. The Aboriginal Advisory Panel also guided the development of the nested study described here.

### 2.3. Setting

The study was conducted in six Aboriginal Medical Services—one urban and five regional—in three Australian states (New South Wales, Queensland, and South Australia).

### 2.4. Procedure

HPs and pregnant women recruited to the ICAN Quit in Pregnancy trial were asked, as part of the consent process, to agree or decline an additional option of audio recording the part of their consultations relating to smoking cessation. Defining the audio recording as an additional option was deemed necessary and more appropriate by the Stakeholder and Consumer Aboriginal Advisory Panel as it was felt that audio recordings might be perceived as more intrusive by Aboriginal women and might deter them from consenting to the ICAN Quit in Pregnancy trial. Taking into consideration that pregnant women discuss multiple pregnancy and nonpregnancy-related issues during their visits to the Aboriginal Medical Service, the Stakeholder and Consumer Aboriginal Advisory Panel found it to be unacceptable to record the entire session in order to extract only the smoking-related counselling. Therefore, the staff was instructed to record only the content regarding smoking during any visit of the recruited pregnant woman, if both HP and the woman had consented to recordings. This was done to encourage pregnant women's consent to the recording and protect privacy for other issues. All content was deidentified regarding the women and HPs. Research facilitators were provided with two audio recorders and were requested to coordinate with the other HP recording of a mix of initial and follow-up consultations (i.e., prequit attempt and postquit attempt up to the 4-week visit) with at least three pregnant women (expected recruitment for the ICAN Quit in Pregnancy trial was 10 eligible consenting women per service) [13], for a total of nine audio recordings per service.

### 2.5. Participants

HPs were eligible if they consulted with pregnant women either for pregnancy confirmation, antenatal care, and/or routine care. Pregnant women were eligible if they were ≤28 weeks of gestation, Aboriginal and/or Torres Strait Islander or expectant mothers of Aboriginal and/or Torres Strait Islander babies, aged ≥16 years old, and smoked tobacco at any level of consumption. Participating women received up to $60 AUD as reimbursement for their time for ICAN Quit in Pregnancy trial-related visits. Women or HPs did not receive additional reimbursement for audio-recording consultations.

### 2.6. Qualitative Interviews

At the end of the ICAN Quit in Pregnancy trial, semistructured interviews were conducted with staff from the services (including managers, research facilitator, and HPs) to assess the acceptability of the study and intervention. No direct questions regarding feasibility and acceptability of audio-recording smoking cessation-related consultations were included due to time constraints. However, a few general questions were included regarding what study procedures worked well and/or what were the more challenging aspects (including “How did the study go for you? What were the best things about the project? What went really well? What were some of the more challenging aspects? How did you manage these challenges? What impact did it have on your daily practice?”).

### 2.7. Analysis

#### 2.7.1. Feasibility Measures

These were collected by the research facilitator, including proportion consenting to audio recording, number of recordings performed, profession of HP (general practitioner/midwife/nurse/Aboriginal health worker) performing the consultation, and date (pre/post intervention).

#### 2.7.2. Qualitative Interviews

Interviews at the end of the ICAN Quit in Pregnancy trial were audio recorded and transcribed. Data relevant to the feasibility and acceptability of audio-recording smoking cessation-related consultations was extracted.

#### 2.7.3. Behaviour Change Techniques

Recordings were transcribed by a professional service and coded independently by two certified BCT coders (YBZ and ES). Patients' responses to the HP were not coded. Coding was based on Michie et al.'s 2010 taxonomy of smoking cessation BCTs v1, which includes the operational definition of each BCT, categorized under BCTs that address motivation, maximizing self-regulatory capacity/skills, promoting adjuvant activities, and general aspects of the interaction focusing on delivery of the intervention, information gathering, and communication [7, 10]. Initial interrater agreement level (% positive agreement) was 48% (calculated by identifying the proportion of all BCTs within a transcript that were recognized by both coders). Discrepancies were resolved through discussion, until agreement reached 100%.

### 2.8. Ethics

The study was approved by the following ethics committees: (1) University of Newcastle Human Research Ethics Committee (HREC) (Reference H-2015-0438), (2) Aboriginal Health & Medical Research Council HREC (Reference #1140/15), (3) South Australia Aboriginal Health HREC (Reference #04-16-652), and (4) Far North Queensland HREC (Reference #16/QCH/34–1040). All participants provided and signed an informed consent to participate in the study.

## 3. Results

### 3.1. Feasibility Measures

**Figure 1:**
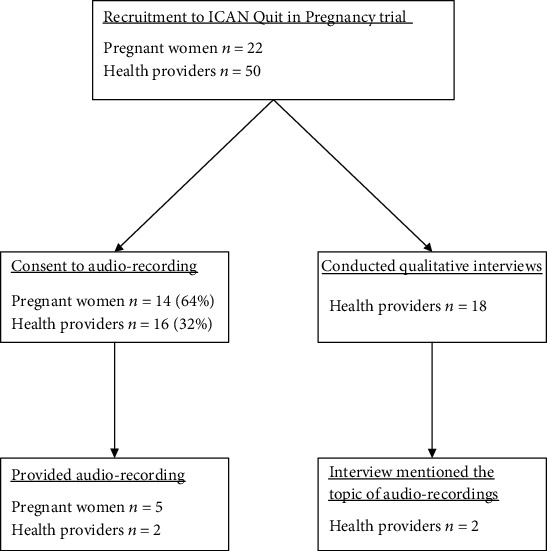
Flowchart of pregnant women and health providers' participation.

In total, 22 pregnant women were recruited to the ICAN Quit in Pregnancy trial; 14 provided consent to audio recordings (64%) (Figure 1). Of the 50 recruited HPs, we have data regarding agreement to audio recording for 23, with 16 providing consent (69.5%, 32% from all HPs). Two Aboriginal Medical Services (of six) provided seven audio recordings, from two HPs and five women, all from the pre-intervention period. One service provided three recordings, with three pregnant women (one recording per woman), all with the same midwife. The second service provided four recordings, with two women (two recordings per woman), all with the tobacco action worker (a HP employed to assist with smoking prevention and cessation activities), who was also the research facilitator.

### 3.2. Qualitative Interviews

A total of eighteen interviews were conducted from all six Aboriginal Medical Services at the end of the ICAN Quit in Pregnancy trial (Figure 1). The topic of audio-recording consultations was discussed in two interviews, one with the research facilitator at a service that provided three audio recordings and another with the manager of a service that did not provide any audio recordings. Women's consent to the audio recordings varied considerably, with one service reporting having no issues “even signing video-voice recording consents, you know, they weren't finicky about it,” and the other service stating none of the women found it acceptable “No, they didn't want it recorded.” In the service that did provide audio recordings, these were still considered a difficult task to remember: “The doctors and midwives quite often forgot to use voice recorder. The GPs didn't remember very often that they had the voice recorders. Obviously very busy as well, so that, you know, it's understandable.”

### 3.3. Behaviour Change Techniques

On average, the two HPs spent two minutes per consultation discussing smoking with the pregnant woman (range 00:47–03:47 minutes). They used few BCTs for each consultation (average 4 BCTs, range 2–8) (Table 1). The most common BCTs used were “building general rapport,” “general communication approaches,” and “information gathering and assessment.” Supplementary Table S1 provides a summary of all of BCTs used, with example quotes.

In one consultation, the HP (tobacco action worker) was passive in response to the woman's enquiry, not taking advantage of her interest to further the discussion around smoking cessation.

Woman: “Hopefully I can quit once the baby is born. I mean I'd rather quit before the baby is born but….” HP: “Yeah well we see how it goes. You seem like you're giving it… something in here might help.” Woman: “Oh is that the smoking program. Smoking cessation program?” HP: “Yes it is.” Woman: “Isn't it kind of like this?” HP: “It's too early to be asking questions.”

## 4. Discussion

### 4.1. Main Findings

In this nested study within six Aboriginal Medical Services, using audio recording of HP-pregnant women's smoking counselling was not feasible as planned. Despite a reasonable rate of agreement to the consultations being recorded, very few recordings were obtained, and all of them in the pre-intervention trial phase (i.e., pre-training). Analysis of the few recordings that were provided showed that HPs devoted little time to smoking cessation support and used few evidence-based BCTs.

### 4.2. Comparison with the Literature

Audio recording of HP-patient visits as a way to assess actual provision of care and communication has been used successfully in the past, including in the context of smoking cessation counselling at specialist services [8, 12, 17]. Lorencatto et al. analysed 15 transcribed audio recordings from three smoking cessation services to develop the original methodology for specifying BCTs in practice [8]. Another study used 34 transcripts of audio recordings from two smoking cessation services to examine fidelity of treatment manuals and actual practice [12]. To the best of our knowledge, these recordings were of the entire patient visit and have not been used specifically with pregnant women or in an Indigenous context. In our study, the entire visit could not be recorded (following the advice of our Stakeholder and Consumer Aboriginal Advisory Panel). This may have reduced the practicality of obtaining audio recordings as HPs needed to stop the visit artificially and remember to start (and stop) recording when discussing smoking.

The initial interrater agreement level was low compared to that reported in other similar studies [8, 12, 17], despite the fact that both coders were trained and certified. This highlights the need for better training and using at least two coders for all transcripts with an iterative process in future studies.

The current Australian guidelines recommend that all HPs ask pregnant women regarding tobacco use, advise pregnant women who smoke to quit smoking and provide information on the most effective methods, and help them to quit by referring them to a Quitline, encouraging them to use behavioural strategies and using nicotine replacement therapy when appropriate [18]. Depending on the time available to the HP, guidelines recommend providing behavioural counselling which can include, among other things, strategies for coping with smoking triggers, addressing barriers to quitting, and setting a quit date [18]. Currently, smoking cessation support during pregnancy is suboptimal [19–21], with previous research suggesting that HPs might be concerned that raising the issue will damage their therapeutic relationship; therefore, HPs put an emphasis on building a positive rapport with the women, being nonjudgmental and supportive [20–22]. The findings from this study provide further support for this as “building general rapport” was one of the most common BCTs used. BCTs that were found in previous research [10, 11, 23] to be associated with cessation success such as “facilitate barrier identification and problem solving,” “facilitate action planning/identify relapse trigger,” and “facilitate goal setting” were not used. Future interventions with HPs should focus on skills training of “how” to assist pregnant women to quit using other BCTs that have been suggested as effective in pregnancy [11, 23, 24], while being nonjudgmental and supportive. The ICAN Quit in Pregnancy training laid emphasis on these skills; unfortunately, no recordings were taken to inform whether the training had been successful or HP fidelity with the approach.

Using more BCTs has been previously suggested to yield better results [23]. Time is perceived as a leading barrier to providing adequate smoking cessation support in primary care [22, 23]. In this study, HPs spent on average only two minutes on their smoking-related consultation. Therefore, it is essential to understand which combination of effective BCTs would be feasible in this busy setting.

### 4.3. Strengths and Limitations

This is the first study that explored smoking cessation-related BCTs used in primary care settings with pregnant Aboriginal and Torres Strait Islander women. It is not possible to generalize the findings from the few audio recordings that were collected. There may have been longer consultations around smoking that included other important issues impacting smoking that women did not want recorded. Other HPs may have used other BCTs or more BCTs. Furthermore, as only pre-training audio recordings were collected, it was not possible to explore whether the intervention had any impact on the length or BCT content of the consultations. Data regarding the reasons why women and/or HPs did not consent to the audio recording, or did not perform the audio recording (for example, whether another person accompanying the pregnant woman was present during the consultation), were not collected, impacting the ability to fully understand the factors influencing the feasibility and acceptability of the audio recording.

### 4.4. Implications for Future Research and Practice

The results of this study will be used to inform a larger cluster randomized controlled study (SISTAQUIT®), in up to 30 Aboriginal Medical Services, which is also attempting to collect audio recordings of smoking-related counselling and compare BCT use both pre- and post-intervention, between the control and intervention groups. Comparing BCT use between different HPs, such as GPs, midwifes, and tobacco action workers, is also important and needs to be taken into consideration when designing future research. Suggested changes to increase the feasibility of obtaining recordings could include the following:

1. Implementing a computerized system to flag participating woman that agreed to be audio recorded, including a reminder at the beginning of the visit
2. Supplying each service with more audio recorders (one for each HP)
3. Providing an incentive for both HP and the women to perform the recording
4. Exploring other ways that might be more feasible and acceptable to the communities such as using observation (without audio recording) by training the research facilitator in BCT identification
5. Using HP self-report of BCTs with a template

## 5. Conclusions

Recording HP consultations with pregnant women about their smoking was not feasible as planned. Analysis of the few recordings that were provided showed that HPs devoted little time to smoking cessation and used few evidence-based BCTs. Future research will need to explore feasible ways to assess BCT use in primary care and specifically in Aboriginal health services, including which BCT combination would be the most effective in time-deprived primary care settings.

## Figures and Tables

**Table 1:** Behaviour change techniques used in audio-recorded consultations as part of ICAN Quit in Pregnancy trial.

Consultation number and time recorded (minutes)	Behaviour change techniques (BCTs) used
Service #1: consultations with midwife	
Patient #1 (02:05)	Six BCTs: (i) RC1: build general rapport (ii) RC2: general practitioner communication approaches (iii) RC6: offer/direct towards appropriate written materials (iv) BM3: provide feedback on current behaviour and progress (v) BM11: measure CO and explain the purposes of CO monitoring (vi) A1: advise on stop smoking medication
Patient #2 (00:48)	Three BCTs: (i) RC7: information gathering and assessment (ii) BM5: provide normative information about others' behaviour and experiences (iii) BM9: facilitate identification of reasons for wanting and not wanting to stop smoking
Patient #3 (03:47)	Four BCTs: (i) RC7: information gathering and assessment (ii) A1: advise on stop smoking medication (iii) A3: ask about experiences of stop smoking medication that the smoker is currently using (iv) BS11: advise on avoiding social cues for smoking

Service #2: consultations with tobacco action worker	
Patient #4 First visit (02:55)	Five BCTs: (i) RC1: build general rapport (ii) RC2: general practitioner communication approaches (iii) RC7: information gathering and assessment (iv) BM3: provide feedback on current behaviour and progress (v) BM7: provide rewards contingent on effort or progress
Patient #4 Second visit (00:47)	Two BCTs: (i) RC1: build general rapport (ii) BM13: create or reinforce negative associations
Patient #5 First visit (01:13)	Three BCTs: (i) RC1: build general rapport (ii) RC7: information gathering and assessment (iii) BM7: provide rewards contingent on effort or progress
Patient #5 Second visit (02:25)	Two BCTs: (i) RC1: build general rapport (ii) RC7: information gathering and assessment

## Data Availability

No data is available.

## Supplementary Materials

Supplementary Materials Table S1: a summary of all of the behaviour change techniques (BCTs) used with examples of quotes.
