# Supplementary material for: Feasibility of Audio-Recording Consultations with Pregnant Australian Indigenous Women to Assess Use of Smoking Cessation Behaviour Change Techniques
Source: J Smok Cessat. 2021 Jan 13;2021:6668748. doi: 10.1155/2021/6668748 (PMC8279198; doi:10.1155/2021/6668748)
Supplement: Supplementary Materials — Table S1: a summary of all of the behaviour change techniques (BCTs) used with examples of quotes. [file 6668748.f1.docx]

**Supplementary Table 1: A summary of all of the behaviour change techniques (BCTs) used with examples of quotes**

| **Behaviour change technique** | **Description of Behaviour change technique** | **Example quotes** | **Overall number of times BCT was used** |
| --- | --- | --- | --- |
| RC1 Build general rapport | Establish a positive, friendly and professional relationship with the smoker and foster a sense that the smoker’s experiences are understood | “we've got some strategies to help with that (smoking cessation” (midwife); “that's a good way to think about it” (tobacco action worker) | 5 |
| RC2 General practitioner communication approaches | Communication that includes one or more of the following: Eliciting and answering questions; Using reflective listening; Summarizing information, and confirming client decisions | “And (name of aboriginal health worker) was saying that that is inspiring you to change your smoking?” (midwife); “So you've sort of gone along the whole reducing (smoking)?” (tobacco action worker) | 5 |
| RC6 Offer/direct towards appropriate written materials | Distinguish what are, and are not, appropriate written materials and offer/direct clients to these in ways that promote their effective use | “… some resources on quitting and deciding to quit and the nicotine options that are there” (midwife) | 1 |
| RC7 Information gathering and assessment | Any information gathering that provides the practitioner with the knowledge needed from the client for appropriate behaviour change techniques to be delivered. Includes one or more of the following: Assessing current and past smoking behaviour; Assessing current readiness and ability to quit; Assessing past history of quit attempts; Assessing withdrawal symptoms; Assessing nicotine dependence; Assessing number of contacts who smoke; Assessing attitudes to smoking; Assessing level of social support; Assessing physiological and mental functioning | “Smoking is currently 15 a day?” (midwife); “You've been thinking about quitting a lot, for a long time?” (midwife); “Have you got other family or friends who smoke around you?” (midwife); “So on average you've been smoking a bit less then?” (tobacco action worker); “So when you were up the hospital you didn't have any smokes?” (tobacco action worker) | 8 |
| A1 Advise on stop smoking medication | Includes one or more of the following: Explaining the benefits of medication, safety, potential side-effects, contra-indications, how to use them most effectively; Advising on the most appropriate medication for the smoker; Promoting effective use; Explaining how to obtain medications, enacting the necessary procedures to ensure the smoker gets their medication easily and without charge where appropriate | “…instructions with Quick Mist Spray and then I've also given directions on how to use it because a lot of people have problems how to lift it up… you can have up to 15 sprays a day and it's 1mg. So that's the lowest one you can get.” (midwife) | 2 |
| A3 - Ask about experiences of stop smoking medication that the smoker is currently using | Asses usage, side effects and benefits experienced of medication that the smoker is currently using | “Are you using any stuff instead of smokes, like any nicotine replacement therapy stuff?” (midwife)  “How did you find it when you tried those (lozenges)? Did you find that you could cut back on the smokes with them?” (midwife) | 1 |
| BM3 Provide feedback on current behaviour and progress | Give feedback arising from assessment of current self-reported or objectively monitored behaviour (e.g. expired-are CO) and/or progress towards becoming a permanent non-smoker | “So for you to come in now and say 'I haven't got any smokes on me and I'm using this e-cig' that's a massive big difference from that first appointment when you came and seen me” (tobacco action worker) | 2 |
| BM5 - Provide normative information about others’ behaviour and experiences | Involves providing information about how the smoker’s experience compares with that of other smokers who are trying to quit, as to indicate that a particular behaviour or sequence of behaviours are common, or uncommon, amongst other smokers trying to quit | “I actually hear a lot of mums say 'When I go out to have a smoke I tell the kids to keep away… And I go out of the house and I have five minutes just to myself.’” (midwife) | 1 |
| BM7 Provide rewards contingent on effort or progress | Give praise or other rewards for the effort the smoker is making in relation to smoking cessation and if the smoker has engaged in activities that aid cessation, such as correct medication use | “That's really good” (in response to woman stating she was trying to reduce) (tobacco action worker) | 3 |
| BM9 - Facilitate identification of reasons for wanting and not wanting to stop smoking | Help the smoker to arrive at a clear understanding of his or her feelings about stopping smoking, why it is important to stop and any conflicting motivations | “It's (smoking) keeping you sane. In what way is it keeping you sane?” (midwife) | 1 |
| BM11 Measure CO and explain the purposes of CO monitoring | Measure expired- air carbon monoxide concentration and explain to the smoker the reasons for measuring CO at different time points (e.g. before and after the quit date) | “…the 23ppm… matches the frequent smoker level… and then the 5.66 FCO is here for the 'mother' column and that's sort of more the addicted smoker. So that's a bit higher, the amount of nicotine that's going through, isn't it?” (midwife) | 1 |
| BM13 -Create or reinforce negative associations | Present descriptions or labels that aim to generate negative emotional associations with smoking other than by providing information about the negative consequences of smoking | “How did you find like people's perceptions and opinions if they saw you when you were pregnant with a smoke? .... The judgmental type thing? …. Have you ever had anybody actually say something to you?” (tobacco action worker) | 1 |
| BS11 - Advise on avoiding social cues for smoking | Give specific advice on how to avoid being exposed to social cues for smoking (e.g. explaining to friends that you have stopped and asking them not to smoke around you) | “So what are you going to do if you go to their house and they're smoking?... if you've got the replacement things in your handbag that might help too?” (midwife) | 1 |
